# Lipid profile frequency and the prevalence of dyslipidaemia from biochemical tests at Saint Louis University Hospital in Senegal

**DOI:** 10.11604/pamj.2014.17.75.3577

**Published:** 2014-01-31

**Authors:** Dominique Doupa, Abdou Salam Mbengue, Fatou Agne Diallo, Modou Jobe, Arame Ndiaye, Adama Kane, Alassane Diatta, Meissa Touré

**Affiliations:** 1 Laboratoire de Biochimie-Biologie Moléculaire Unité de formation et de recherche (UFR), Santé Université Gaston Berger, Saint Louis, Sénégal; 2 Laboratoire de Bactériologie-Virologie, CHU Aristide Le Dantec, Dakar, Sénégal; 3 Laboratoire de Biochimie-Biologie Moléculaire, Faculté de Médecine, de Pharmacie et d'Odontologie de l'Université Cheikh Anta Diop, Dakar, Sénégal; 4 Service de cardiologie, CHU Aristide Le Dantec, Dakar, Sénégal

**Keywords:** Lipid profile, dyslipidaemia, prevalence, Senegal

## Abstract

**Introduction:**

The aim of this study was to evaluate the frequency of lipid profile requests and the prevalence of dyslipidemia in patients at the biochemistry laboratory of St. Louis University Hospital, as well as their correlation with sex and age.

**Methods:**

This was a retrospective study reviewing 14,116 laboratory results of patients of both sexes, over a period of six months (January-June 2013) regardless of the indication for the request. The lipid parameters included were: Total cholesterol, HDL-cholesterol, LDL-cholesterol, triglycerides with normal values defined as follows: Total cholesterol (<2g/l), HDL- cholesterol (>0,40g/l), LDL- cholesterol (<1,30g/l) and Triglycerides (<1,50g/l).

**Results:**

The average age of our study population was 55.15 years with a female predorminance (M/F = 0.60). The age group most represented was that between 55-64 years. The frequency of lipid profile request in our sample was 9.41% (or 1,329). The overall prevalence of isolated hypercholesterolemia, hyperLDLaemia, hypoHDLaemia, hypertriglyceridaemia, and mixed hyperlipidemia were respectively 60.91%, 66.27%, 26.58%, 4.57% and 2.75%. Hypercholesterolemia, hyperLDLaemia, hypertriglyceridaemia and mixed hyperlipidaemia were higher in women with respectively 66.22%, 67.98%, 4.58%, 2.89% than in men (52.01%, 62.81%, 4.44% and 2.40% respectively). On the other hand, the prevalence of hypoHDLaemia was higher in males (32.19%) compared to females (23.76%). Hypercholesterolemia correlated significantly with age and sex.

**Conclusion:**

Our study showed a relatively low request rate for lipid profile and a high prevalence of dyslipidaemia hence the importance of conducting a major study on the prevalence of dyslipidaemia and associated factors in the Senegalese population.

## Introduction

In Africa, death from chronic noncommunicable diseases (NCDs) and its complications far exceed that of AIDS, malaria and tuberculosis combined [1]. It is estimated that over the next decade, these will be higher than those for all communicable diseases [2]. Among the chronic NCDs, cardiovascular diseases remain the leading cause of death and disability in developed countries, and increasingly in developing countries. They are a major cause of premature mortality, morbidity and high healthcare costs. They have an important impact in terms of public health and most of them are the result of clinical complications secondary to atherosclerotic lesions of the arteries. Among the primary risk factors, the Framingham study has shown the important role of lipids and especially of hypercholesterolaemia in causing lesions [3]. In Senegal, as in most of sub-Saharan Africa, there are few data on the epidemiology of dyslipidaemia across the population. The objectives of this study were to evaluate the frequency of requests for lipid profile and the prevalence of dyslipidaemia in patients at the biochemistry laboratory of St. Louis University Hospital in Senegal from January to June 2013.

## Methods

This was a retrospective study conducted from January to June 2013 from laboratory requests and involving 14, 116 patient results at the biochemistry laboratory of St. Louis University Hospital (a national referral hospital) regardless of the indication for the biochemical tests. Patients whose results were included in the laboratory register were included regardless of the number of lipid parameters that were included in the analysis report.

Serum assays for lipid parameters such as total cholesterol, HDL-cholesterol and triglycerides were performed by standard enzymatic methods using an automatic multiparametric analyser (Cobas Integra, Roche) and BTS-310 (Biosystems) and the results expressed in (g/l). Serum concentrations of LDL-cholesterol were obtained by calculation according to the Friedewald method [4]. When triglyceride levels exceeded 3.4 g/l, LDL was not determined. The standards held in this laboratory are therefore high and meeting those held internationally.

### Operational definition of variables

The values used to define dyslipidemia are those of the National Educational Program (NECP) [5]. Hypercholesterolaemia: Total cholesterol >2g/l (5.2mmol/L). HypoHDLaemia: HDL-Cholesterol <0.40g/l (1.04mmol/L). Hypertriglyceridaemia: Triglycerides >1.50g/l (1.69mmol/L). Mixed hyperlipidaemia: Total cholesterol >2g/l (5.2mmol/L) and triglycerides >1.50g/l (1.69mmol/L). HyperLDLaemia: LDL-cholesterol >1.3g/l (3.1mmol/L)

### Statistical analysis

All data were analyzed with Epi Info7 software (CDC, Atlanta). Quantitative variables were expressed as mean with standard deviation. Qualitative variables were expressed as numbers and percentages. The chi-square test and Fisher's exact test were used to explain the relationship between two variables. The difference was considered to be statistically significant when p <0.05. All analysis were performed with a 95% confidence interval.

## Results

**Figure 1:**
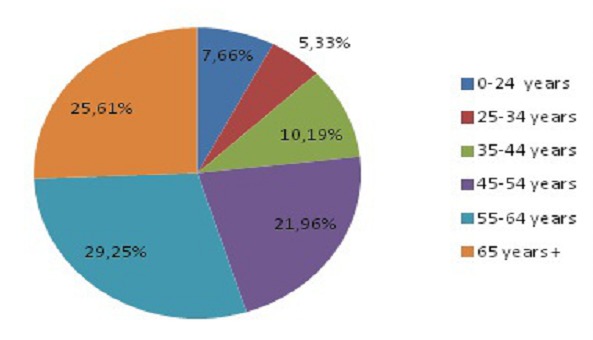
Population distribution according to age

**Figure 2:**
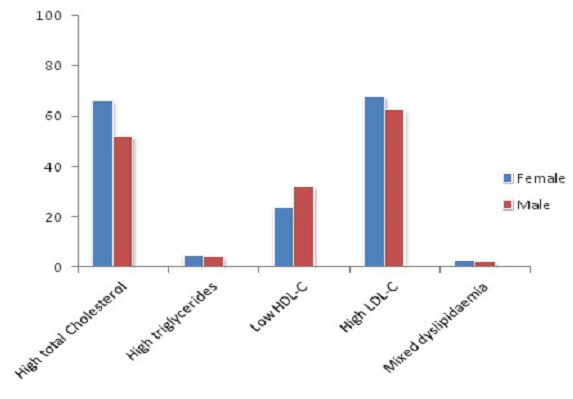
Distribution of dyslipidaemia according to sex

1329 patients were included in our study including 499 men (37.5% of the sample) and 830 women (62.5% of the sample) giving a sex ratio (M/ F) of 0.60. The average age of the population was 55.15 years, ranging from 2 to 96 years. The general characteristics of the study population are summarized in Table 1. The age group most represented was that between 55 to 65 years (Figure 1). In our study population, 9% (1329/14116) had at least a request for lipid profile as part of the biochemical investigations. The respective prevalence of hypercholesterolaemia, hyperLDLaemia, HypoHDLaemia hypertriglyceridaemia and mixed hyperlipidaemia were 60.91%, 66.27%, 26.58%, 4.57% and 2.75% (Table 2). Hypercholesterolemia, hyperLDLaemia, hypertriglyceridaemia and mixed hyperlipidaemia were higher in women with respectively 66.22%, 67.98%, 4.58%, and 2.89% compared to men with respectively 52.01%, 62.81%, 4.44% and 2.40%. On the other hand, the prevalence of HypoHDLaemia was greater in males (32.19%) than females (23.76%) (Figure 2).

**Table 1:** General characteristics of the population studied

Variables	Total	Hypercholestaerolemia	Hypertriglyceridaemia	Low HDL	High LDL	Mixed Dyslipidaemia
**Sex**						
Males	499	259(52.1)	18(4.44)	131(32.19%)	255(62.81%)	12(2.4)
Females	830	547(66.2%)	35(4.58%)	182(23.76%)	516(67.98%)	24(2.89%)
**Age (years)**						
0-24	82	31 (37.8)	2 (6.9%)	9(31,03%)	17 (2.71%)	1 (1.22%)
25-34	57	31 (56.36)	1 (2.04%)	12(24%)	32 (5.1%)	0 (0%)
35-44	109	67 (62.4)	8 (7.84%)	29(28,43%)	65 (10.35%)	6 (5.5%)
45-54	235	165 (70.2)	9 (3.93%)	52(22,61%)	159 (25.32%)	6 (2.55%)
55-64	313	193 (61.6)	17 (5.63%)	89(29,7%)	201 (32.01%)	11 (3.51%)
65+	274	154 (56.2)	8 (3.14%)	74(29%)	154 (24.52%)	5 (1.82%)

**Table 2:** Distribution of patients according to the type of dyslipidaemia

Dyslipidaemia	Number	Percentage (%)
Hypercholesterolaemia	815	60.9
HyperLDLaemia	780	66.2
HypoHDLaemia	315	26.5
Hypertriglyceridaemia	54	4.5
Mixed hyperlipidaemia	37	2.7

Table 3 shows the risk factors associated with the types of dyslipidaemia. Hypercholesterolaemia, hyperLDLaemia and hypoHDLaemia correlated significantly with sex (p < 0.05). Hypercholesterolaemia was the only type that significantly and positively correlated with age in this study.

**Table 3:** Relationship with dyslipidaemia with age and sex

Variables	Total	Hypercholesterolaemia	Hypertriglyceridaemia	Low HDL	High LDL	Mixed Dyslipidaemia
**Sex**						
Males	499	259 (52.1)	18 (4.4%)	131 (32.1%)	255(62.8%)	12 (2.4%)
Females	830	547 (66.2%)	35 (4.5%)	182 (23.7%)	516(67.9%)	24 (2.8%)
Total	**1329**	806 (60%)	54 (4.5%)	315 (26.5%)	780(66.2%)	37 (2.7%)
*P value*		<0.01	0.08	<0.01	0.02	0.6
**Age (years)**						
≥ 50	761	475 (62.4%)	33 (4.5%)	204 (28.02%)	478 (65.84%)	21 (2.7%)
< 50	309	166 (54.2%)	12 (5%)	61 (25.42%)	150 (63.29%)	8 (2.5%)
Total	1070	641 (60%)	45 (46%)	265 (27.3)	628 (65.2%	29 (2.7%)
*P value*		0.01	0.75	0.43	0.47	0.87

## Discussion

Senegal, like most developing countries is experiencing an epidemiological transition. The latest statistics released by the demographic and health survey (EDSIV 2005) [6] showed that 22% of women in Senegal have a high BMI being either overweight or obese. This study which was aimed at determining the frequency of request for lipid profile and the prevalence of dyslipidaemia over a six-month period showed 1329 patients out of 14,116 (being 9% of requests) having biochemical test for lipid profile from the University Hospital of Saint-Louis. In a similar study conducted at the biochemistry laboratory of the University Hospital of Cocody in Ivory Coast, Tiahou G et al in 2010 [7] found a frequency of 5.7% of request for lipid profile. In this study, this low prescribing of lipid profile was attributed partly to the low qualification level of prescribers and also the lack of proper equipments suitable for the production of certain specialised exams. This relatively higher frequency of requests for lipid profile in our study is due in large part to the existence of a specialized care unit for cardiovascular disease and qualification level of prescribers (a cardiologist and two internists).

In socio-demographic terms we noted a female predominance (sex ratio male / female) 0.60, slightly higher than the last census in St. Louis [8]. This imbalance could be explained by the large male migration to the capital [8]. The average age of our study population was 55.15 ± 16.2 years. Two other studies have found similar results including that of Essais et al in the Tunisian population [9] and that of Yousef in the Jordanian population [10].

Our study revealed a high prevalence of dyslipidaemia. Of a total of 1329 patients, 60.91% had hypercholesterolemia, 66.27% with hyperLDLaemie, 26.58% with hypoHDLaemie, 4.57% with hypertriglyceridaemia and 2.75% with mixed hyperlipidaemia. Furthermore, hypercholesterolaemia, hypoHDLaemia, hypertriglyceridaemia and mixed hyperlipidaemia were higher among females compared to their male counterparts. On the other hand hypoHDLaemia was higher in men. A study conducted in Jordan by Khader et al in 2010 [10] found a prevalence of 48.3% of hypercholesterolemia, 40.1% of HypoHDLaemia, 40.7% of HyperLDLaemia, 43.6% of Hypertriglyceridaemia. According to these authors the prevalence of dyslipidaemia except for hypercholesterolaemia was higher among women than among men. Our results are also similar to those of Pessinaba et al in 2013 [11] in the population Dagana (Senegal) where a high prevalence of dyslipidemia was also found in women.

In our study, hyperLDLaemia was the most common lipid abnormality. This result is similar to that of Erem C et al in Turkey [12] who found a predominance of hyperLDLaemia in the study population. On the other hand, Tiahou G et al in their study found hypercholesterolemia as the most lipid abnormality.

Our study also found that hypercholesterolaemia was significantly associated with age and sex (p <0.01 Table 3). A recent study conducted in Pakistan by Zahid N et al [13] showed that all dyslipidaemia except hypoHDLaemia correlated statistically significant to age and sex. Similar results were reported by Erem et al in Turkey. However, in our study only hypercholesterolaemia was significantly associated with age and sex. This slight difference may be explained in part by the inclusion criteria for patients, since in these two studies, only patients aged 25 years and older were included but then shows the weakness of our sampling.

## Conclusion

This study revealed a high prevalence of dyslipidaemia despite low requesting for lipid profile at Saint Louis University Hospital. A national survey is needed to better assess the prevalence of dyslipidemia in the Senegalese population. Meanwhile, awareness of risk is necessary and should lead to the establishment of a prevention program.
